# A new species of bromeliad-feeding *Cephaloleia* Chevrolat (Coleoptera, Chrysomelidae, Cassidinae) from Costa Rica: evidence from DNA barcodes, larval and adult morphology and insect diets

**DOI:** 10.3897/zookeys.477.8220

**Published:** 2015-01-26

**Authors:** Carlos García-Robledo, Charles L. Staines, W. John Kress

**Affiliations:** 1 Principal affiliation: Department of Multi-trophic Interactions, Institute of Ecology (INECOL), Xalapa, Mexico; 2 Department of Entomology, National Museum of Natural History, Smithsonian Institution; 3 Department of Botany, National Museum of Natural History, Smithsonian Institution

**Keywords:** Braulio Carrillo National Park, Bromeliaceae, *Cephaloleia kuprewiczae*, COI DNA barcode, *Pitcairnia arcuata*, *Pitcairnia brittoniana*

## Abstract

The Neotropical genus *Cephaloleia* Chevrolat (Coleoptera: Chrysomelidae: Cassidinae) includes 214 species distributed from the south of Mexico to Argentina. *Cephaloleia* beetles feed mostly on plants from the order Zingiberales. The interactions between *Cephaloleia* beetles and their Zingiberales host plants is proposed as one of the oldest and most conservative associations. Here we describe a new species of *Cephaloleia* (*Cephaloleia kuprewiczae* **sp. n.**) that feeds on two species of bromeliads (*Pitcairnia arcuata* and *Pitcairnia brittoniana*, Bromeliaceae: Pitcairnioideae). *Cephaloleia kuprewiczae* was previously described as *Cephaloleia histrionica*. This study includes evidence from DNA barcodes (COI), larval and adult morphology and insect diets that separates *Cephaloleia kuprewiczae* from *Cephaloleia histrionica* as a new species.

## Introduction

The Neotropical genus *Cephaloleia* Chevrolat (Coleoptera: Chrysomelidae: Cassidinae) includes 214 species distributed from the south of Mexico to Argentina (Staines and García-Robledo 2014). *Cephaloleia* beetles are also known as the “rolled-leaf beetles” because larvae and adults of the majority of *Cephaloleia* species feed on the scroll formed by the young leaves of their hosts. *Cephaloleia* beetles feed mostly on plants from the order Zingiberales. The interactions between *Cephaloleia* beetles and their Zingiberales host plants is one of the oldest and most conservative insect-host plant associations (García-Robledo and Staines 2008).

Two species of *Cephaloleia* are known to complete their life cycle on plants in the families Arecaceae and Orchidaceae (Urueta-Sandino 1972, Sekerka et al. 2013). Here we describe *Cephaloleia kuprewiczae* sp. n., a new species of *Cephaloleia* from a tropical montane forests in Costa Rica that feeds on plants from the family Bromeliaceae.

Individuals of this species were previously treated as *Cephaloleia histrionica* Baly (García-Robledo et al. 2013a). Combining DNA barcodes, records on host use and larval and adult morphologies, the objective of this study is to describe this new species and clarify the species delimitations between *Cephaloleia histrionica* and *Cephaloleia kuprewiczae* sp. n.

## Materials and methods

### Study site and species of interest

**Figure 1.**
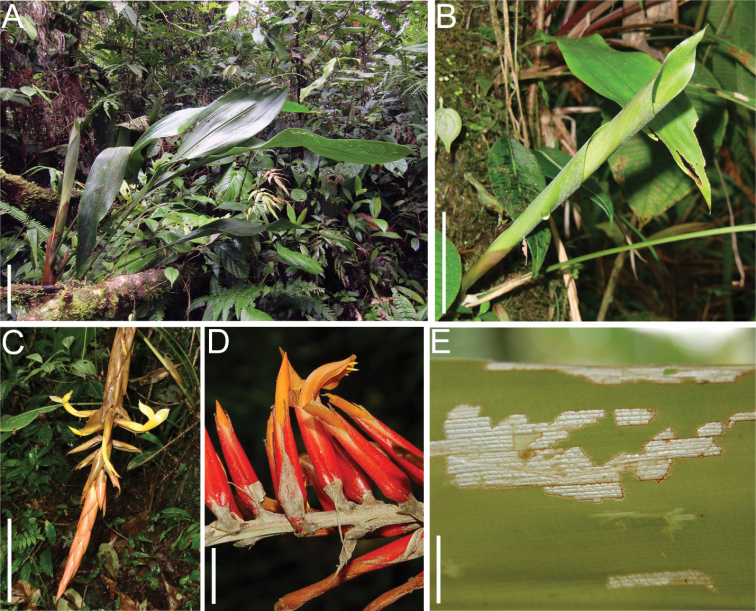
*Pitcairnia arcuata* and *Pitcairnia brittoniana* (Bromeliaceae), host plants of *Cephaloleia kuprewiczae*. **A** *Pitcairnia arcuata*, habit **B** Detail of a rolled leaf used as a larval and adult food source and adult oviposition site **C** *Pitcairnia arcuata* Inflorescence **D** *Pitcairnia brittoniana* inflorescence **E** Leaf damage produced by a feeding adult *Cephaloleia histrionica*. Scale bars: **A–C** = 10 cm; **D, E** = 1 cm. Modified from García-Robledo et al. 2013a.

This research was conduct at two localities in Costa Rica, Central America. Larvae and adults of *Cephaloleia kuprewiczae* sp. n. were collected in Costa Rica, Heredia Province at the Braulio Carrillo National Park and the Selva Tica and Rara Avis hotels, two private properties that abut this National Park. Additional material was collected at 1500 m elevation in the Braulio Carrillo National Park (Selva Tica: 10°18'10"N, 84°02'02"W Rara Avis: 10°16'54"N, 84°02'41"W, Braulio Carrillo 1500 m elevation shelter: 10°14'32"N, 84°02'58"W). The life zones in this study area include tropical premontane and montane forests (García-Robledo et al. 2013a). Larvae were collected from rolled leaves of *Pitcairnia arcuata* (André) André and *Pitcairnia brittoniana* Mez (Bromeliaceae) (Figure 1).

In addition, we collected larvae and adults of *Cephaloleia histrionica* at two localities in the Talamanca Cordillera in Costa Rica, near the border with Panama. We selected these localities because they are the closest forests in Costa Rica to the type locality of *Cephaloleia histrionica* (Syntype examined: Panama, Province of Chiriquí, District of Bugaba, 652 m. elevation Champion [printed label]/ Paratipo [handwritten red label]/ F. Monros Collection 1959) (Staines and García-Robledo 2014). The first locality in the Talamanca Cordillera was a tropical rain forest at 60 m.a.s.l. in the Pacific slope, at 27 km from the locality where the type specimen was collected (Costa Rica, Puntarenas Province, Ciudad Neilly, 8°38'56"N, 82°56'43"). Additional surveys were performed at Las Cruces Biological Station, a Tropical Premontane Forest at 1200–1500 m.a.s.l. The distance of Las Cruces Biological Station to the locality where the type specimen of *Cephaloleia histrionica* was collected is ca. 38 km (Costa Rica, Puntarenas Province, Cotobrus region 8°47'07"N, 82°57'31"). All individuals were collected from *Costus laevis* Ruiz & Pav., *Costus guanaiensis* Rusby and *Dimerocostus strobilaceus* O. Kuntze (Costaceae).

Individuals were collected in ET-OH 95% for further morphological descriptions and DNA analyses. For adults, measurements were taken with an ocular micrometer. Pronotal length and width were taken along the midlines. Elytral width was measured at the humerus. Elytral length was measured from the base to the apex. Total length was measured from the base of the antennae to the apex of the elytra. For larva descriptions, measurements were taken with an ocular micrometer or from scanning electron microscope images. Total larval length was measured from the anterior to the posterior margins. Total width was measured at the widest point.

### DNA sequencing and differences in COI sequences between *Cephaloleia kuprewiczae* and *Cephaloleia histrionica*

Legs of each adult and larval tissue were placed in 96-well plates. DNA extractions were performed following the protocols described in García–Robledo et al. (2013b). Amplification of the mitochondrial gene cytochrome oxidase COI was conducted in 96-well plate formats using the COI Folmer primer (García–Robledo et al. 2013b). PCR was followed by ExoSap purification. Amplified products were subjected to standard sequencing using BigDye Di-Deoxy terminator sequencing. Sequences were aligned using multiple sequence alignment with high accuracy and high throughput.

To estimate the similarity of COI sequences among individuals of *Cephaloleia kuprewiczae* sp. n. and *Cephaloleia histrionica*, we generated a neighbor-joining tree, estimating bootstrap support after 100 replicates. Analyses were performed using Geneious Pro V 5.6.5 (Biomatters-development-team 2012). Differences among COI sequences were estimated as the percentage of bases/residuals that are identical (DNA sequences: GenBank, accession No. KC794541–KC794652 and Suppl. material 1).

## Results

### Host plants of *Cephaloleia kuprewiczae* sp. n. and *Cephaloleia histrionica*

We recorded two host plant species for *Cephaloleia kuprewiczae* sp. n. At 700 m.a.s.l., larvae and adults of this species feed inside the scroll formed by the young rolled leaves of *Pitcairnia arcuata* (Figure 1A–C). At 1500 m.a.s.l., *Cephaloleia kuprewiczae* sp. n. feeds on *Pitcairnia brittoniana* (Figure 1D). The damage produced by this herbivore differs from the typical longitudinal strip mining damage described for other *Cephaloleia* beetles (García-Robledo and Staines 2008) (Figure 1E).

*Cephaloleia histrionica* was recorded feeding only on plants from the family Costaceae. In the tropical rain forest at Talamanca (60 m.a.s.l.), this species was collected from *Costus guanaiensis*. In the tropical premontane forest (1200 m.a.s.l.) this species was recorded feeding on *Costus laevis* and *Dimerocostus strobilaceus*.

### Species description

#### Cephaloleia kuprewiczae

Taxon classificationAnimaliaColeopteraChrysomelidae

García-Robledo & Staines sp. n.

http://zoobank.org/14DE2AAF-3973-4279-8E09-41E7024C51D3

##### Material.

Holotype (male), ♂, ‘COSTA RICA: Heredia, Braulio Carrillo Nat. Park, near Rara Avis Hotel | 700 m | 9°17'N, 84°03'W | 25 November 2011 | Carlos García-Robledo | K1163_EK-25-nov-2011-12 | *Pitcairnia arcuata* (André) André’ (USNM). Paratypes (9 males): with same label data as holotype (USNM, INBIO).

##### Differential diagnosis.

**Figure 2.**
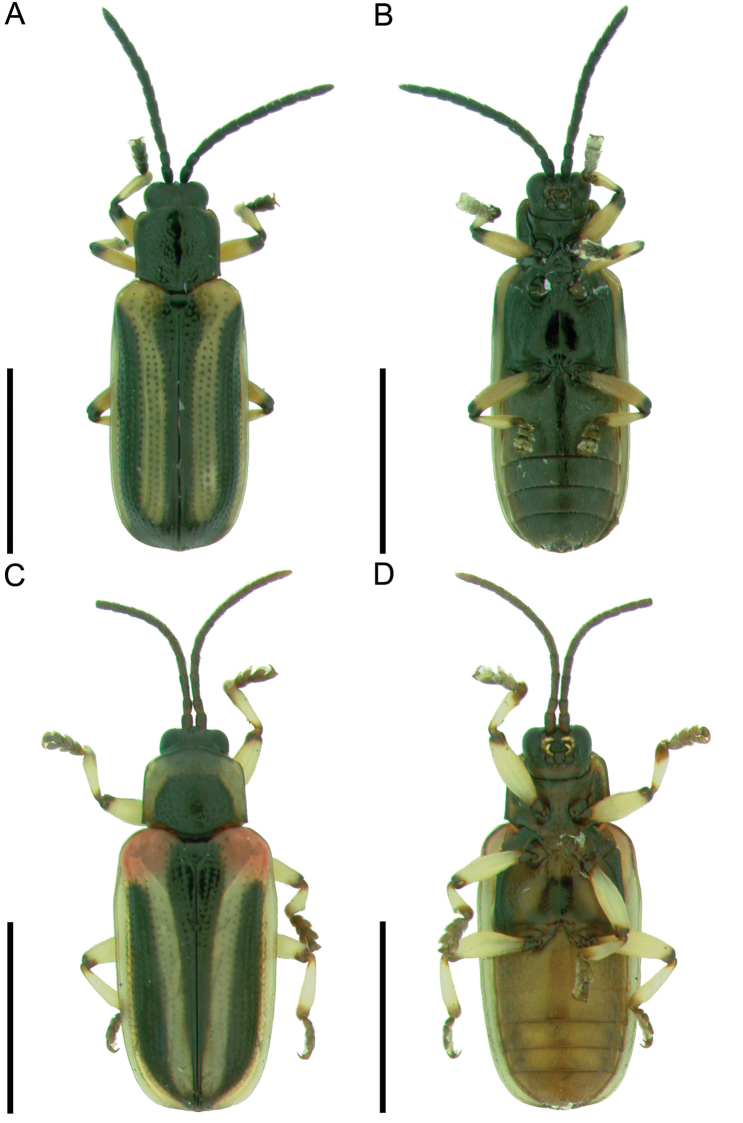
**A–B** *Cephaloleia kuprewiczae* **A** dorsal view **B** ventral view **C–D** *Cephaloleia histrionica* **C** Dorsal view **D** Ventral view. Scale bars = 3 mm.

*Cephaloleia kuprewiczae* sp. n. is most similar to *Cephaloleia histrionica* and in some degree to *Cephaloleia semivittata* Baly. It can be easily distinguished from *Cephaloleia semivittata* by its larger size, the elytral declivity beginning at puncture row 7, by antennomere 2 being ¾ the length of 1, by the depressed vertex of the head, and by the medial longitudinal impunctate area on the pronotum. It can be distinguished from *Cephaloleia histrionica* by its rectangular shape and black pronotum (Figure 2). The suture between abdominal sterna 1 and 2 being obsolete medially, by elytral puncture row 10 being near lateral margin, by antennomere 2 being cylindrical, by the humerus not being reddish, and by the sinuate lateral margins of the pronotum.

##### Description.

Elongate; parallel-sided; subdepressed; head, antennae, and scutellum brownish-black; pronotum brownish-black with yellow lateral margins; elytra yellow with brownish-black sutural and subhumeral vittae; venter brownish-black with lateral margins of abdominal sterna paler; legs yellowish with tibio-femoral joint and tarsi brownish (Figure 2A). **Head:** vertex densely punctate, depressed between eyes; medial sulcus absent; keel present between antennal bases; clypeus punctate, with fringe of setae on anterior margin. **Antenna:** reaches beyond humerus; filiform; antennomere 1 subincrassate; 2 cylindrical, ¾ length of 1; 3 cylindrical, subequal in length to 1; 4 to 10 cylindrical, decreasing in length; 11 1½× length of 10, pointed at apex; 1 to 4 punctate; 5 to 10 setose. **Pronotum:** longer than wide; lateral margin sinuate, canaliculate; anterior angle rounded, not produced; anterior margin curved forward; posterior angle acute; posterior margin bisinuate; surface irregularly punctate except impunctate medial longitudinal line from base to apex. **Scutellum:** pentagonal; alutaceous (Figure 2A). **Elytron:** lateral margin straight, smooth; exterior apical angle rounded, smooth; apical margin rounded, smooth; sutural angle without tooth; humerus rounded, not produced, impunctate; with 10 regular rows of punctures plus scutellar row; with declivity beginning behind humerus at puncture row 7 (Figure 2A). **Venter:** pro-, meso-, and metasterna impunctate medially, punctate laterally; abdominal sterna finely punctate, each puncture with pale seta; suture between abdominal sterna 1 and 2 obsolete medially; apical margin of last sternite notched in male (Figure 2B). **Leg:** long, slender; punctate; tibia with fringe of setae on inner apical margin. **Total length:** 5.0 to 5.7 mm.

##### Host plants.

*Pitcairnia arcuata* (André) André and *Pitcairnia brittoniana* Mez (Bromeliaceae) (Figure 1).

##### Etymology.

Named for Erin K. Kuprewicz, who discovered this species and its interaction with *Pitcairnia* (Bromeliaceae) host plants. The name is feminine.

### Description of *Cephaloleia kuprewiczae* sp. n. and *Cephaloleia histrionica* immature stages

#### *Cephaloleia kuprewiczae* sp. n.

**Figure 3.**
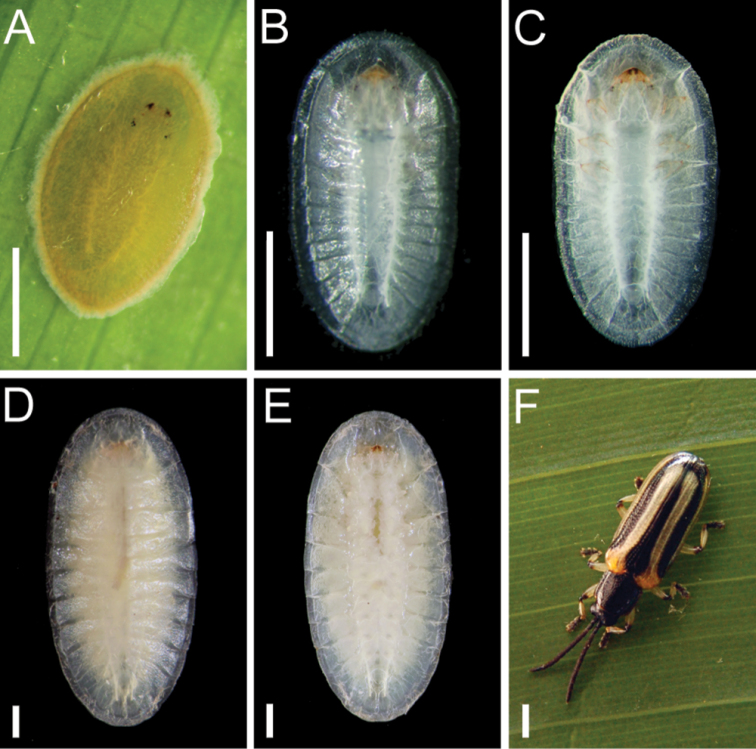
**A** Egg **B–C** First instar larva (dorsal and ventral views) **D–E** Second instar larva (dorsal and ventral views). **F** Adult *Cephaloleia histrionica*. Scale bars in all panels = 1 mm. From García-Robledo et al. 2013a.

**Figure 4.**
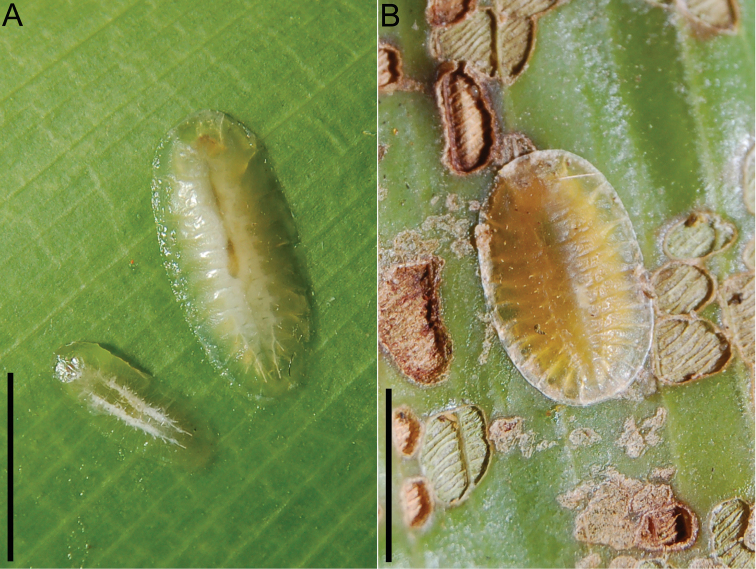
Larvae of *Cephaloleia kuprewiczae* (**A**) and *Cephaloleia histrionica* (**B**) feeding on their host plants. Scale bars = 3 mm.

*Cephaloleia kuprewiczae* immature stages were previously described as *Cephaloleia histrionica* (García-Robledo et al. 2013a). *Cephaloleia kuprewiczae* eggs are pale yellow (Figures 3A). The attachment tissue of the egg to the substrate is pale cream colored. Eggs were found attached to the inner surfaces of rolled leaves of *Pitcairnia arcuata* and *Pitcairnia brittoniana*. Mean egg length ± SD = 2.55 ± 0.09 mm, mean width ± SD = 1.59 ± 0.10 mm, *n* = 6. Larva color when alive is creamy-white becoming translucent laterally and apically, with some yellowish areas medially (Figures 3B–E, 4A). Color when fixed in EtOH is yellowish-brown. Dorsum without medial setose ridge. Total length: 8.6–9.3 mm; width 4.6–4.9 mm (*n* = 4).

**Figure 5.**
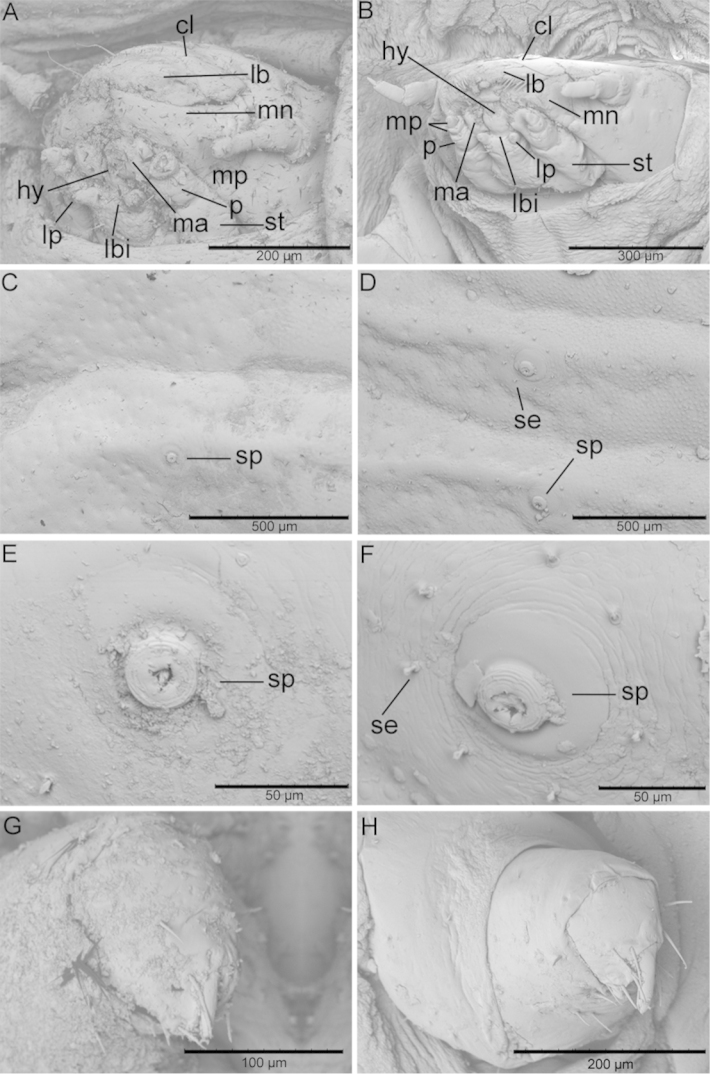
Differences in microstructures between larvae of *Cephaloleia kuprewiczae* (left column) and *Cephaloleia histrionica* (right column). **A–B** Head **C–D** Details of dorsal papillae and spiracles **E–F** Detail of spiracle and surrounding setae **G–H** Front leg. cl: clypeus, hy: hypopharynx, lb: labrum, lbi: labium, lp: labial palp, ma: malum, mn: mandibula, mp: maxillary palp, p: palpifer, se: seta, sp: spiracle, st: stipe.

Dorsum. Pronotum without raised central area; micropustulate (Figure 5C); with pale setae along lateral and apical margins; lateral and apical margins with numerous shallow sulci (Figure 5C). Mesonotum without raised central area or carina or sulcus; micropustulate; laterally with numerous shallow sulci on expansion. Metanotum with central portion micropustulate; without carina or sulcus. Abdominal tergites 1–6 slightly narrowed in middle; without carina laterally; spiracle near basal margin; each spiracle appears as spot with darker margin, orifice (Figure 5E). Abdominal tergites 7–10 without surface plicae or carinae.

Venter. Surface of expansions smooth, sulcate laterally. Head with surface sparsely punctate, without setae; clypeus smooth, without setae; labrum with 6 long and 6 short setae on apical margin, with four large punctures each with a single seta; mandibles tridentate (Figure 5A); maxillary palps with 2 palpomeres, each palpomere with 3 setae and 8 sensilla setae at apex; mala robust, clavate, with fringe of long setae at apex; labium smooth (Figure 5A). Antenna with 3 antennomeres; antennomere 1 short, robust, ½ length of 2; 2 cylindrical, longer than 1 and 3 combined; 3 shortest, with ring of 19 setae at apex (Figure 5A). Prosternum longer than others, wider than long, slightly depressed in middle; surface rugose-striate. Meso- and metasterna wider than long, slightly depressed in middle; surface rugose-striate. Abdominal sternites 1–8 wider than long, decreasing in width; with transverse sulcus just beyond middle and second transverse sulcus near apex; sterna 9–10 fused, rounded at apex. Leg stout; coxa with 10 setae; femur wider and longer than tibiotarsus; tibiotarsus subconical, with a robust claw and 6 setae at apex (Figure 5G).

#### Cephaloleia histrionica

Color when alive yellow-white becoming translucent laterally and apically, with some yellowish areas medially (Figure 4B). Color when dead yellowish-brown. Dorsum without medial setose ridge. Total length: 8.6–9.3 mm (n=4); width 4.6–4.9 mm.

Dorsum. Pronotum without raised central area; micropustulate; with pale setae along lateral and apical margins; lateral and apical margins with numerous shallow sulci (Figure 5D). Mesonotum without raised central area or carina or sulcus; micropustulate; laterally with numerous shallow sulci on expansion. Metanotum with central portion micropustulate; without carina or sulcus. Abdominal tergites 1–6 slightly narrowed in middle; without carina laterally; spiracle near basal margin; spiracles appear as spot with darker margin, orifice surrounded by five setae as in Figure 5F. Abdominal tergites 7–10 without surface plicae or carinae.

Venter. Surface of expansions smooth, sulcate laterally. Head with surface sparsely punctate, without setae; clypeus smooth, without setae; labrum with 10 long and 6 short setae along apical margin, with four large punctures each with a single seta; mandibles tridentate (Figure 5B); maxillary palps with 2 palpomeres, each palpomere with 3 setae and 8 sensilla at apex; mala robust, clavate, with fringe of long setae at apex; labium smooth (Figure 5B). Antenna with 3 antennomeres; antennomere 1 short, robust, ½ length of 2; 2 cylindrical, longer than 1 and 3 combined; 3 shortest, with ring of 19 setae at apex (Figure 5B). Prosternum longer than others, wider than long, slightly depressed in middle; surface rugose-striate. Meso- and metasterna wider than long, slightly depressed in middle; surface rugose-striate. Abdominal sternites 1–8 wider than long, decreasing in width; with transverse sulcus just beyond middle and second transverse sulcus near apex; sterna 9–10 fused, rounded at apex. Leg stout; coxa with 4 rows of 2 setae each; femur wider and longer than tibiotarsus, with 8 setae; tibiotarsus subconical, with a strong claw and 6 setae at apex (Figure 5H).

#### Differential diagnosis for larval stages

Larvae of *Cephaloleia kuprewiczae* sp. n. and *Cephaloleia histrionica* display obvious differences in shape and color (Figures 4 and 5). Larvae of *Cephaloleia kuprewiczae* sp. n. are elongated and white (Figure 4A), while *Cephaloleia histrionica* larvae are more rounded and yellow (Figure 4B). The head of *Cephaloleia kuprewiczae* sp. n. is rounded (Figure 5A), the head of *Cephaloleia histrionica* larvae are flattened (Figure 5B). The setae along dorsal ridges are absent in *Cephaloleia kuprewiczae* sp. n. larvae (Figure 5C) but present in *Cephaloleia histrionica*. Larvae of these species are also easily differentiated by a series of five setae surrounding each spiracle only present in *Cephaloleia histrionica* (Figure 5E–F).

### DNA barcode divergence between *Cephaloleia kuprewiczae* sp. n. and *Cephaloleia histrionica*

**Figure 6.**
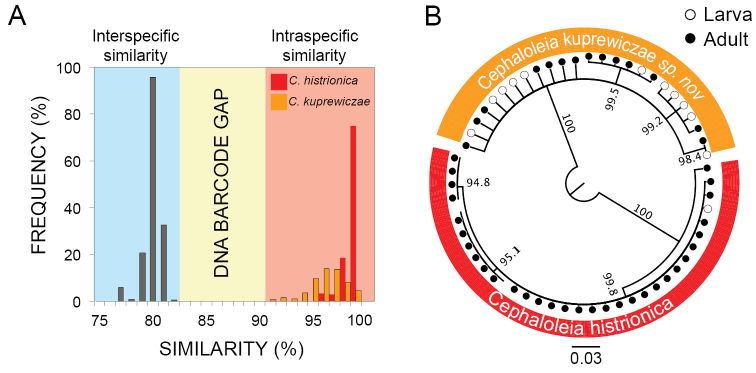
**A** Frequency distributions of inter and intraspecific similarities for beetle COI sequences (paired comparisons, percentage of bases/residuals that are identical for each comparison for cytochrome oxidase I (COI) sequences included in Figure 6B). **B** Identification of *Cephaloleia kuprewiczae* sp. n. and *Cephaloleia histrionica* using cytochrome oxidase I (COI) sequences. Neighbor-joining tree includes bootstrap values (%) supporting species identifications. Filled circles represent DNA sequences obtained from adults. Empty circles represent DNA sequences obtained from larvae.

Within-species similarities of COI sequences ranged between 91–100% (Figure 6A). Similarities of COI sequences between species ranged between 77–82% (Figure 6A). These two species can be unambiguously identified as they display a DNA barcode gap between 83–90% (Figure 6A).

Using the DNA barcode COI, we correctly identified the species of all larvae included in this study (Figure 6B). The neighbor-joining tree assigned all *Cephaloleia kuprewiczae* sp. n. individuals to one group. COI sequences of *Cephaloleia histrionica* from the population in the tropical rain forest (60 m.a.s.l.) and premontane forest (1200 m.a.s.l.) in the Talamanca Cordillera are similar and were assigned to one group (Figure 6B).

## Discussion

This study combined morphological, ecological and molecular evidence to discover a new species. Larval morphology and differences in host plant orders are strong evidence that these are two different species. Molecular analyses confirmed that this complex includes at least two different species. It is important to note that with this information, we were able to reassess adult morphologies of *Cephaloleia kuprewiczae* sp. n. and *Cephaloleia histrionica* adults, finding obvious morphological differences between these two species (Figure 2).

Previous studies reported two species of *Cephaloleia* completing their life cycles on palms and orchids. *Cephaloleia vagelineata* Pic larvae and adults were recorded on *Elaeis guineensis* Jacq., *Corozo oleifera* (H.B.K.) Bailey, *Cocos nucifera* L. (Urueta-Sandino 1972) and *Astrocaryum chonta* Matrius (Couturier and Kahn 1992) (Arecaceae). *Cephaloleia orchideivora* Sekerka et al. larvae and adults feed on Elleanthus cf. robustus (Rchb. f.) Rchb. f., *Elleanthus* sp., *Epidendrum werklei* Schltr., *Oerstedella exasperata* (Rchb. f.) Hágsater, and *Oerstedella wallisii* (Rchb. f.) Hágsater (Orchideaceae) (Sekerka et al. 2013).

*Cephaloleia kuprewiczae* sp. n. is a third example of diet expansion beyond the order Zingiberales in rolled-leaf beetles. Further studies are required to determine if other *Cephaloleia* species are also adapted to other non-Zingiberales host plants.

## Supplementary Material

XML Treatment for Cephaloleia kuprewiczae

## Supplementary materials

Supplementary material 1 DNA barcodes (COI) for specimens of *Cephaloleia histrionica* and *Cephaloleia kuprewiczae* included in this study. Data type: Fasta file. Explanation note: Single-line descriptions for each sequence include: Collection number, *Cephaloleia* species, host plant species, elevation and locality. DNA sequences from type specimens include the tag genseq-1 in the description line. This dataset is made available under the Open Database License (http://opendatacommons.org/licenses/odbl/1.0/). The Open Database License (ODbL) is a license agreement intended to allow users to freely share, modify, and use this Dataset while maintaining this same freedom for others, provided that the original source and author(s) are credited.Carlos Garcia-Robledo, Charles L. Staines, W. John Kress
